# Stem Cell Growth and Differentiation in Organ Culture: New Insights for Uterine Fibroid Treatment

**DOI:** 10.3390/biomedicines10071542

**Published:** 2022-06-29

**Authors:** Ana Salas, Silvia Beltrán-Flores, Carmen Évora, Ricardo Reyes, Francisco Montes de Oca, Araceli Delgado, Teresa A. Almeida

**Affiliations:** 1 Department of Biochemistry, Microbiology, Cell Biology and Genetics, Biology Section, Science Faculty, University of La Laguna, Ave. Astrofísico Fco. Sánchez s/n. San Cristóbal de La Laguna, 38200 Santa Cruz de Tenerife, Spain; asalaspe@ull.edu.es (A.S.); alu0101115443@ull.edu.es (S.B.-F.); rreyesro@ull.edu.es (R.R.); 2 Institute of Tropical Diseases and Healthcare of the Canary Island, Ave. Astrofísico Fco. Sánchez s/n. San Cristóbal de La Laguna, 38200 Santa Cruz de Tenerife, Spain; 3 Department of Chemical Engineering and Pharmaceutical Technology, Faculty of Pharmacy, University of La Laguna, Ave. Astrofísico Fco. Sánchez s/n. San Cristóbal de La Laguna, 38200 Santa Cruz de Tenerife, Spain; cevora@ull.edu.es (C.É.); adelgado@ull.edu.es (A.D.); 4 Institute of Biomedical Technologies (ITB), Medicine Section, Faculty of Health Science, University of La Laguna, St. Santa María Soledad, s/n. San Cristóbal de La Laguna, 38200 Santa Cruz de Tenerife, Spain; 5 Hospital Quironsalud, St. Poeta Rodríguez Herrera 1, 38200 Santa Cruz de Tenerife, Spain; fmontesdeoca@ginefem.com

**Keywords:** uterine leiomyoma, myometrium, fibroids, organ culture, surface markers, stem cells, cell repopulation, mediator complex subunit 12 (MED12), high mobility group AT-hook 2 (HMGA2)

## Abstract

Organ culture allows for the understanding of normal and tumor cell biology, and tissues generally remain viable for 5–7 days. Strikingly, we determined that myometrial and *MED12* mutant leiomyoma cells repopulated cell-depleted tissue slices after 20 days of culture. Using immunofluorescence and quantitative PCR of stem cell and undifferentiated cell markers, we observed clusters of CD49b^+^ cells in tumor slices. CD49b^+^ cells, however, were sparsely detected in the myometrial slices. Almost all LM cells strongly expressed Ki67, while only a few myometrial cells were stained for this proliferation marker. The CD73 marker was expressed only in tumor cells, whereas the mesenchymal stem cell receptor KIT was detected only in normal cells. HMGA2 and CD24 showed broader expression patterns and higher signal intensity in leiomyoma than in myometrial cells. In this study, we propose that activating CD49b^+^ stem cells in myometrium leads to asymmetrical division, giving rise to transit-amplifying KIT^+^ cells that differentiate to smooth muscle cells. On the contrary, activated leiomyoma CD49b^+^ cells symmetrically divide to form clusters of stem cells that divide and differentiate to smooth muscle cells without losing proliferation ability. In conclusion, normal and mutant stem cells can proliferate and differentiate in long-term organ culture, constituting a helpful platform for novel therapeutic discovery.

## 1. Introduction

Uterine leiomyoma (LM), also called fibroid, is the most common gynecological tumor in women of reproductive age, with an incidence of 40% at the age of 35 and nearly 70–80% by the onset of menopause [1]. Although mostly benign, severe symptoms develop in 15–30% of patients; irregular, prolonged, or heavy vaginal bleeding are the most common manifestation frequently associated with anemia [1]. In addition, leiomyomas may interfere with natural and in vitro fertility and increase the risk of adverse pregnancy outcomes [2].

Fibroids are monoclonal tumors raised from smooth muscle cells (SMC) that require the presence of multipotent somatic stem cells (SSC) for in vivo growth [3]. LM stem/progenitor cells isolated by Hoechst dye efflux assay expressed the cell surface marker CD49b [4]. Isolated CD49b^+^ cells expressed high levels of stem cell markers such as OCT4, KLF4, and NANOG. In addition, CD49b^+^ cells formed in vitro colonies and achieved tumor-regeneration in vivo, strongly supporting CD49b^+^ cells as stem cells [4]. Furthermore, cells isolated using CD34 and CD49b cell surface markers coupled to microarray gene expression profiling suggested a hierarchical differentiation pattern, whereby CD34^+^/CD49b^+^ stem cells (6%) first differentiate to CD34^+^/CD49b^−^ (7%) intermediary cells, which terminally differentiate to CD34^−^/CD49b^−^ cells (87%). Authors proposed that stem cells undergo asymmetric division, giving one daughter cell that retains self-renewal ability while the other intermedia cell is destined to differentiate into an SMC [5]. Alternatively, the CD24 surface marker is more highly expressed in fibroid smooth muscle cells (CD24^hi^) than normal myometrium [6]. CD24^hi^ cells display features of progenitor cells such as reduced expression of the alpha-smooth muscle actin and strong expression of the SSC surface marker CD73.

The acquisition of a genetic hit may transform a myometrial stem cell into a leiomyoma tumor-initiating cell [3]. Two driver genes, *HMGA2* (high mobility group AT-hook 2) and *MED12* (mediator complex subunit 12), were found to be altered in 90% of fibroids [7]. HMGA2 is widely restricted to the embryonic stage and decreases to undetectable levels in adult tissues [8]. High expression of *HMGA2* has been frequently detected in fibroids compared to matched myometrium [9,10]. Mutations in *MED12* hot spot exon 2 have been found in 44–70% of uterine leiomyomas from Caucasian, Black, and Asian women [11,12,13,14].

Although fibroids are steroid hormone-dependent tumors, stem cells express very low levels of estrogen and progesterone receptors (ERα and PGR, respectively). As stem cells differentiate to more mature stages, steroid receptor expression increases [4,15]. Therefore, leiomyoma SSC must establish paracrine interactions with other cell populations responsible for mediating steroid hormone action [15]. In this regard, organ culture allows the cells to communicate and respond to environmental cues while maintaining the original extracellular matrix (ECM). We have previously set up an organ culture of fibroids where LM slices maintained their structural integrity and functional activity for 7 days in culture [16]. Subsequent cell death led to dramatic cell reduction in the tissue sections. Surprisingly, after 20 days of culture, cells repopulated almost the entire tumor slice. This unexpected finding was also observed in myometrium culture. This cell repopulation capacity prompted us to determine the mechanisms behind normal myometrium homeostasis and the growth of fibroids.

Cell characterization in long-term culture demonstrated the different mechanisms of normal and tumor stem cells for repopulation tissue slices, making long-term organ culture a novel therapeutic discovery tool for uterine fibroids.

## 2. Materials and Methods

### 2.1. Tissue Sampling

In total, 4 female patients aged 44–50 years with menorrhagia due to LM admitted to Hospital QuironSalud between 2018 and 2019 were enrolled in this study. The 4 tumors analyzed were intramurals, up to 5–12 cm in size. Three matched myometrial samples were also obtained. All patients were Caucasian and underwent hysterectomy without any previous hormonal treatment for at least 3 months. After surgery, samples were immediately submerged in sterile Hank’s balanced salt solution (HBSS) supplemented with 0.25 μg/mL amphotericin B, 100 U/mL penicillin, and 100 μg/mL streptomycin (Sigma-Aldrich Co., Madrid, Spain). Tissue pieces were processed under sterile conditions within 4 h after surgery.

### 2.2. Tissue Slice Culture

Fresh tissue sections of 500 µm were obtained using a vibratome and cultured as previously described [16]. Two slices were harvested before culture (T0), and two additional slices were collected after 20, 23, 26, and 29 days of culture. For molecular analysis, one slice was submerged in RNAlater and kept at −20 °C until processed, while the other was formalin-fixed for histologic analysis.

### 2.3. Nucleic Acid and Protein Isolation

Tissue slices were placed on lysis matrix D tubes (MP-Biomedicals, Illkirch-Graffenstaden, France) containing 500 μL of Tritidy G (PanReac AppliChem, Barcelona, Spain), homogenized using FastPrep 5 G (2 cycles of 6 m/s for 30 s; MP Biomedicals, Santa Ana, CA, USA) and RNA was isolated following manufacturer instructions. Residual genomic DNA was removed by incubating the RNA samples with RNase-free DNase I and RNasin (Promega Corp., Madison, WI, USA).

### 2.4. Quantitative Polymerase Chain Reaction (qPCR) 

Retro-transcription was carried out as previously described [16]. For qPCR, each sample was analyzed in triplicate in a total reaction volume of 5 μL consisting of a 2.25 μL 12-fold dilution of cDNA, 1× qPCRBIO SyGreen Mix Lo-Rox (PCRBiosystems, London, UK) and 0.25 μM of each primer. A Light-Cycler 480 real-time PCR detection system apparatus was used to quantify all transcripts.

The cycling conditions were 95 °C for 15 min followed by 40–45 cycles of 95 °C for 10 s, 60 °C for 20 s and 72 °C for 30 s. For each experiment, a non-template reaction was included as a negative control. Primers sequences are provided in Table S1.

Plates data were imported into the GenEx 6 data analysis software (MultiD Analyses AB, Gothenburg, Sweden). Data were represented as individual triplicate runs and as average triplicates (with outliers excluded). Grubbs test was used to remove outliers from each group. The relative expression of each gene was calculated using the 2^−ΔΔCt^ method normalized to expression levels of two reference genes (PUM1 and XIAP) stably expressed [17,18]. Log2 transformation of fold-change was used for statistical analysis.

### 2.5. MED12 Mutation Detection

To check for mutations in cDNA, we used a primer pair located in exon 1 and exon 2 (Table S1), covering the hot spot region where 99% of mutations have been described [11]. Sequencing reactions were performed for both strands at the University of La Laguna (SEGAI).

### 2.6. Histological Staining and Immunostaining

Leiomyoma and myometrium samples were fixed in 10% buffered formalin, then embedded in paraffin, and cut in 4 μm-thick sections. The sections were deparaffinized, hydrated, and hematoxylin-eosin (H&E) stained to assess the morphological integrity of tissue samples.

Immunofluorescence (IF) analysis was performed as described previously [19]. Briefly, 3 μm sections were incubated overnight in a humidified chamber at room temperature with primary antibody (Table S2). After washing, Alexa-Fluor488 anti-rabbit IgG or Alexa-Fluor594 anti-mouse IgG (Table S2) were added, and sections were incubated for 1 h protected from light. Finally, samples were counterstained with 1 µg/mL DAPI (Sigma-Aldrich, Madrid, Spain) for 2 min and mounted using Fluoromount-G^TM^ mounting media (ThermoFisher Scientific, Waltham, MA, USA).

Sections were sequentially incubated with two primary antibodies raised in different host species and secondary antibodies tagged with different fluorophores for colocalization analysis. Secondary antibody only (no primary antibody) controls were used to confirm the specificity of the immunostaining. Sections were examined under a fluorescence microscope (Leica DM4000B, Leica Microsystems, Wetzlar, Germany), and images were acquired using a digital camera (Leica DFC300FX). Images obtained from the different fluorescent channels were analyzed using the Image J program, version 1.5a software (NIH). Image J software was used to quantify the proportion of fibroblast and SMC in the slices. First, the total cell area was defined by selecting the green staining, excluding cells from blood vessels and performing color threshold. Similarly, the area occupied by SMC was defined by selecting the red staining corresponding to desmin expressing cells. Finally, the fibroblast area was produced by subtracting the SMC area from the total cell area. Three fields covering the entire slice piece were taken under a fluorescence microscope (Leica DM4000B, Leica Microsystems, Wetzlar, Germany) at a final magnification of X20. The final value was obtained by averaging the three fields.

### 2.7. Statistical Analysis

GraphPad Prism v. 8.0 (GraphPad Software, La Jolla, CA, USA) was used for all statistical analyses. qPCR data were analyzed using a paired t-test (two-tailed) after assessing that the datasets passed the normality tests. When the dataset did not pass the normality test, a two-tailed Wilcoxon matched-pairs signed-ranks test was used. The proportions of SMC and FB were analyzed using a one-way ANOVA with Friedman correction for non-parametric analysis, followed by a Dunnett’s multiple comparison test to compare means for different times to that of the baseline group (T0). For all analyses, *p* < 0.05 was considered to be statistically significant.

## 3. Results

### 3.1. Spontaneous Growth of Myometrial and Leiomyoma Cells

In our previous LM organ culture, cell viability was maintained for one week [16], with almost no cells observed on day 15 (Figure S1A). Surprisingly, from day 20 to 29, tumor cells repopulated the tissue explant (Figure S1A). We then asked whether normal cells could proliferate equally well under similar conditions. Interestingly, the same results were observed in myometrium organ culture (Figure S1B). At longer incubation times (30–35 days), no cells were observed, and the ECM remained the main component of the tissue slices (not shown).

**Figure 1:**
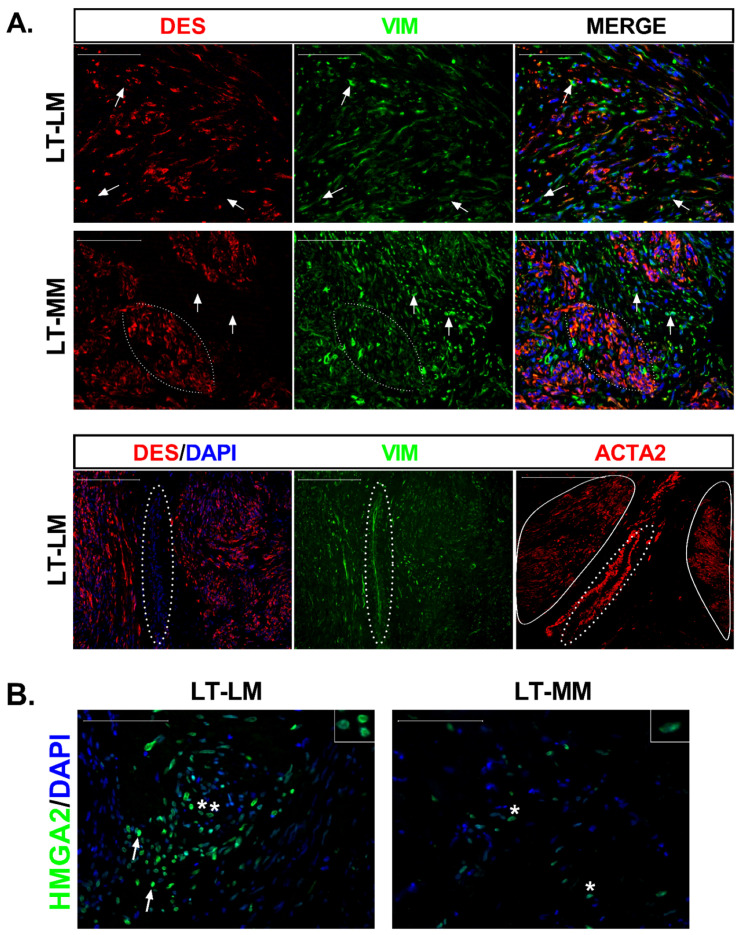
Characterization of cells that repopulated leiomyoma slices and expression of HMGA2 protein. (**A**) Tissue distribution of smooth muscle cells (SMC), fibroblasts (FB), and myofibroblasts at long-term culture (LT) of leiomyoma (LM) and myometrium (MM) slices. DES (red) stains SMCs, whereas VIM (green) stains both SMCs and FBs. DAPI stained cell nucleus (blue). Arrows in DES image point to the DES negative but VIM positive cells. Most of the cells in the reconstituted slices were DES^+^/VIM^+^ (Merge), with few fibroblasts (DES^−^/VIM^+^) dispersed among them (white arrows). Scale bar, 100 µm. The lower images show the colocalization of DES (red) and VIM (green) in an LM section and immunostaining with ACTA2 antibody in the next section. Myofibroblasts (DES^−^/VIM^+^/ACTA2^+^; dashed circle) were detected between two smooth muscle bundles (continuous circle). (**B**) Immunostaining of tissue sections with HMGA2 (green) demonstrated cells with strong (white arrows) and weak (asterisks) signals in LM slices at long-term culture (LT). MM slices showed fewer immunostained cells with lower HMGA2 expression (asterisks). The inserts (top right) show the nuclear immunoreaction. Scale bar, 100 µm.

Both fibroids and myometrium showed smooth muscle cells (DES^+^/VIM^+^) that seemed to spread over the areas previously identified as smooth muscle bundles, with fibroblasts (DES^−^/VIM^+^) mainly localized between them (Figure 1A). In one LM at T20, we detected DES^−^/VIM^+^/ACTA2^+^ cells between SMCs bundles (Figure 1A), indicating the presence of myofibroblasts in the long-term culture of a LM slice. Still, none of the DES^−^ cells in myometrium explants showed immunoreaction for ACTA2 (data not shown).

We observed large differences in the cell coverage between different tumor and myometrial cultured slices. Despite this, the proportion of fibroblast remained similar to T0 (Figure S2A) in cultured normal and tumor slices. SMC slightly decreased after long-term culture compared to T0 but remained greater than fibroblasts at all time points (Figure S2A) except at T23 in LM, where both cell types were in a similar proportion.

### 3.2. MED12 Mutation and HMGA2 Expression Were Maintained in Long-Term Cultures

cDNA sequencing demonstrated the presence of 2-point mutations, 1 deletion coupled with a single point mutation, and 1 insertion/deletion in *MED12* (Figure S3). After long-term culture, preferential expression of the mutant allele was observed in the four tumors analyzed, indicating that the mutations remained during the entire culture period (Figure S3).

RT-PCR and immunohistochemistry showed no *HMGA2* transcript or protein in fibroids and myometria at T0 (data not shown). At long incubation times, HMGA2 immunoreactivity (HMGA2-ir) was observed in the nuclei of myometrial cells in localized areas of the tissue slice. In contrast, a stronger signal and more HMGA2 positive cells were observed in LM slices (Figure 1B). These results agree with qPCR data, showing increased *HMGA2* mRNA expression in LM (161-fold *p* < 0.001) and myometrium (29-fold, *p* < 0.0001) compared to T0 (Figure S4A) as well as in leiomyoma compared to myometrium (7-fold, *p* < 0.001). HMGA2 is an upstream regulator of the transcription factor PLAG1, frequently overexpressed in benign adipocytic tumors [20]. We observed significant upregulation of *PLAG1* in tumor (4-fold, *p* < 0.01) and normal slices (4.6-fold, *p* < 0.001) at long-term culture (Figure S4A).

### 3.3. Differential Expression of Stem Cell and Undifferentiated Cell Markers in Normal and Tumor Slices

**Figure 2:**
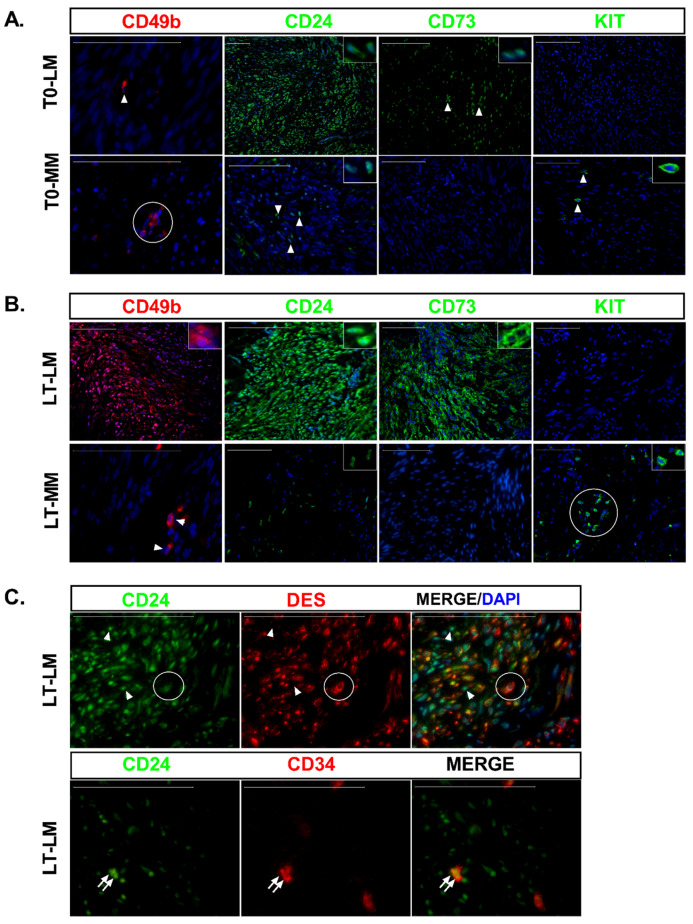
Expression of stem cell and undifferentiated surface markers in leiomyoma and myometrial slices at baseline and long-term culture. (**A**) IF staining of CD49b (red), CD24 (green), CD73 (green), and KIT (green) in leiomyoma (LM) and myometrium (MM) at baseline (T0). DAPI stained cell nucleus (blue). Detailed immunostaining is depicted in the insert. Arrowhead indicates immunostained cells. (**B**) Same marker described in (**A**) at long-term culture (LT). Detailed immunostainings are depicted in the inserts. Arrowhead indicates the CD49b^+^ cells detected in the myometrium. KIT staining in myometrium was found dispersed or in grouped cells (circle). In all sections, nuclei were counterstained with DAPI (blue). (**C**) Extensive colocalization of CD24 (green) with DES (red) in long-term culture slices was observed in LM slices (circle cells), but CD24^+^/DES^−^ cells were also detected (arrowheads). Colocalization of CD24 (green) and CD34 (red) in two LM cells (arrow). Scale bar, 100 µm.

The CD49b^+^ stem cells appeared scattered throughout the myometrium slices at any time point analyzed (T0 and long-term culture), similar to LM at T0 (Figure 2). In these slices, CD49b^+^ cells seemed to be isolated or forming tiny clumps of cells, which were not superior to 3–4 cells, and were always surrounded by unstained cells. On the contrary, in two LM slices at T20, we found clusters formed by hundreds of cells with solid red immunostaining associated with weaker stained cells but still expressing the CD49b stem cell marker (Figure 2B). We did not find CD49b cells in these two tumors at longer incubation times (23, 26, and 29 days of culture). Similarly, we did not find CD49b immunostained cells in the remaining two tumors after long-term culture. This data suggests different mechanisms of stem cell activation in tumor and normal cells. In addition, earlier activation of tumor stem cells may explain their absence in two tumors and longer than 20 days of incubation.

Significant upregulation of *CD49b* mRNA was detected in normal (10-fold, *p* < 0.001) and tumor tissue (334-fold, *p* < 0.001) compared with the corresponding T0 (Figure S4B). However, a comparison of LM and myometrial slices at long-term culture showed significant downregulation of *CD49b* mRNA in leiomyoma cells (4-fold; *p* < 0.01, Figure S4B).

CD24 was barely expressed within localized areas in myometrial slices, while higher CD24 expression levels and a greater cell number were detected in LM slices at T0 (Figure 2A). At long incubation times, most of the cells that repopulated the entire tissue slice were strongly immunostained for CD24 in LM and to a much lesser extent in the myometrium (Figure 2B). Accordingly, significant upregulation of *CD24* mRNA was observed in cultured LM compared to both T0 (7-fold, *p* < 0.001, Figure S4B) and cultured myometrial slices (13-fold, *p* < 0.0001, Figure S4B). Although CD24 colocalized with desmin (CD24^+^/DES^+^) in most cells at long-term culture, few CD24 positive cells did not express desmin (Figure 2C).

**Figure 3:**
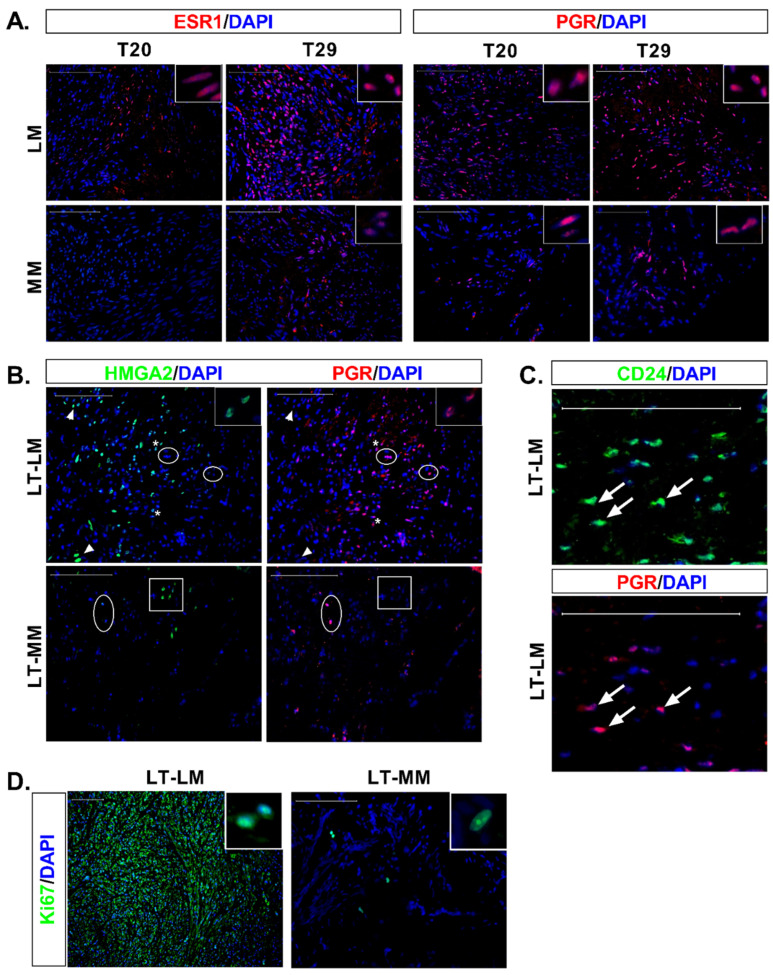
Expression of the ovarian hormone receptors, colocalization of progesterone receptor with HMGA2 and CD24 and expression of Ki67 proliferation marker in long-term culture. (**A**) IF staining of tissue sections with ESR1 (red) and PGR (red), shows more immunoreactive cells at T29 than T20 in LM and MM. A higher number of immunoreactive cells were detected in LM than MM at both time points and for both receptors. (**B**) Colocalization of HMGA2 (green) and PGR (red) in LM at long-term culture (LT). A high HMGA2 signal was detected in cells not expressing PGR (arrowheads), while cells expressing high levels of PGR lacked HMGA2 expression (circle). In cells with weaker expression of HMGA2 and PGR, colocalization between both markers was detected (asterisk, with a detailed view in the insert). In myometrium (MM), cells expressing HMGA2 at variable degree (square) did not express PGR. Similarly, cells expressing PGR (circle), did not express HMGA2. (**C**) Strong colocalization of CD24 (green) with PGR (red) was detected in LM cells (arrow) after long-term culture. (**D**) Almost all cells in the LM slice were Ki-67^+^, whereas few cells were immunostained in MM. The inserts (top right) show a detail of the cell nucleus in both tissues Nuclei were counterstained with DAPI (blue). Scale bar, 100 µm.

Only a few dispersed CD34-ir normal and tumor cells were detected at long-term cultures (data not shown). Endothelial cells from blood vessels express the CD34 surface marker, but no signal for CD24 was seen in these cells (Figure S2B). In addition, we observed blood vessels only at T0 but not after long-term culture. Interestingly, we could find colocalization of CD24 and CD34 in a few cells from two LM slices (Figure 3B). This data supports CD34^+^/CD24^+^ cells as the intermediary CD49b^−^/CD34^+^ SMC isolated previously in fibroid tissues [4]. *CD34* mRNA expression was decreased in LM slices compared to myometrium slices at T20-29 (132-fold, *p* < 0.0001, Figure S4B).

At T0, the *CD73* stem cell surface marker was observed in a few cells in tumor slices, but no immunostaining was detected in the myometrium (Figure 2A). An increased number of immunostained cells was observed in LM slices at long-term culture, with a strong signal detected in the cell membrane (Figure 2B). However, no immunostained cells were detected in the myometrium (Figure 2B). Accordingly, significant upregulation of *CD73* mRNA was observed in LM when compared to both T0 (4-fold, *p* < 0.001) and myometrial slices (2-fold, *p* < 0.001) (Figure S4B).

Striking differences were observed between normal and tumor cells for proto-oncogene KIT. At T0 and long-term culture, few dispersed or grouped KIT-ir cells were detected only in the myometrium (Figure 2A,B). In agreement with this, significant upregulation of *KIT* mRNA was detected in myometrium at T20-29 compared to T0 (4-fold, *p* < 0.001, Figure S4B), and decreased expression was detected in LM compared to myometrium slices at long-term culture (27-fold, *p* < 0.0001, Figure S4B).

### 3.4. Expression of Ovarian Steroids Receptors Was Induced at Long Incubation Times

Immunofluorescence analysis showed that the expression of the ovarian receptors increased gradually over time in both LM and myometrial slices. Thus, at T20, we could not detect ERα in normal cells while few cells positively stain in LM slices (Figure 3A). At T29 greater number of ERα-ir cells were detected in both tissues (Figure 3A). PGR-ir cells were already observed in normal cells at T20, and LM slices showed a greater number of PGR-ir cells compared to myometrial tissue at T20 and T-29 (Figure 3A). This data suggest that the expression of PGR precedes ERα during normal and tumor cell differentiation.

No colocalization of HMGA2 (Figure 3B) or CD24 (data not shown) with PGR was seen in myometrial cells. On the contrary, simultaneous detection of HMGA2-ir or CD24-ir with PGR was observed in LM cells (Figure 3B,C).

Loss of *KLF11* expression is associated with increased PGR signaling and proliferation of leiomyoma cells [21]. We observed decreased expression of *KLF11* mRNA in long-term fibroid slices (3-fold, *p* < 0.001, Figure S4C) that was accompanied by increased levels of *PGRA* mRNA when compared to T0 (2-fold *p* < 0.001). In both tissues, downregulation of *PGRB* (2.5-fold *p* < 0.001, 3-fold *p* < 0.05 for myometrium and leiomyoma, respectively) and *ESR1* mRNA (9-fold *p* < 0.001, 3-fold *p* < 0.01, for myometrium and leiomyoma, respectively) was observed when compared to T0. However, when LM slices were compared to myometrial slices, upregulation of *ESR1* mRNA (10-fold, *p* < 0.0001) was observed in tumor cells (Figure S4C).

### 3.5. Massive Cell Proliferation in Fibroids Contrast with Few Proliferating Cells in Myometrial Slices

To determine cells actively proliferating in tissue slices, we used the Ki67 marker, considered a nuclear antigen only detected in dividing cells [22]. Few Ki67-ir cells were observed in T0 in normal and tumor tissue, according to the low mitotic index frequently observed in these tissues (data not shown). Myometrium showed Ki67 nuclear signal at long incubation times in a few round grouped cells, while most of the leiomyoma cells were strongly stained for the Ki67 proliferation marker (Figure 3D).

### 3.6. Differential Expression of IGF Family in Normal and Tumor Slices

IGF family members have been widely related to LM proliferation and growth. In leiomyoma, most of the IGF members analyzed at long-term cultures showed increased expression compared to T0, being *IGF1* (3-fold, *p* < 0.01), *IGF1R* (5-fold, *p* < 0.001), *IGF2R* (4-fold, *p* < 0.05), *IGFBP2* (5-fold, *p* < 0.001), *IGFBP3* (1600-fold, *p* < 0.001) and *IGFBP6* (2-fold, *p* < 0.05) mRNA significantly upregulated (Figure S4D). In myometrium, only *IGFBP1* (7-fold, *p* < 0.05.) and *IGFBP3* (8-fold, *p* < 0.001) increased expression significantly while *IGFBP5* (2-fold, *p* < 0.01) showed a significant downregulation (Figure S4D). *IGF2* decreased expression was detected in normal (5.5-fold, *p* < 0.01) and tumor tissue slices (3-fold, *p* < 0.001) compared to T0 (Figure S4D).

Comparison of normal and tumor tissue at long-term culture showed increased expression of *IGF1R* (5-fold, *p* < 0.0001) and *IGFBP6* (3.5-fold, *p* < 0.01), whereas *IGFBP1* (4-fold, *p* < 0.05), *IGFBP3* (2.5-fold, *p* < 0.01) and *IGFBP4* (4-fold, *p* < 0.01) were downregulated in tumor slices (Figure S4D).

## 4. Discussion

In this study, we demonstrated the ability of myometrial and leiomyoma stem cells to repopulate tissue slices after long-term organ culture, providing an invaluable model system to investigate the molecular mechanisms behind normal and tumor stem cell proliferation and differentiation.

**Figure 4:**
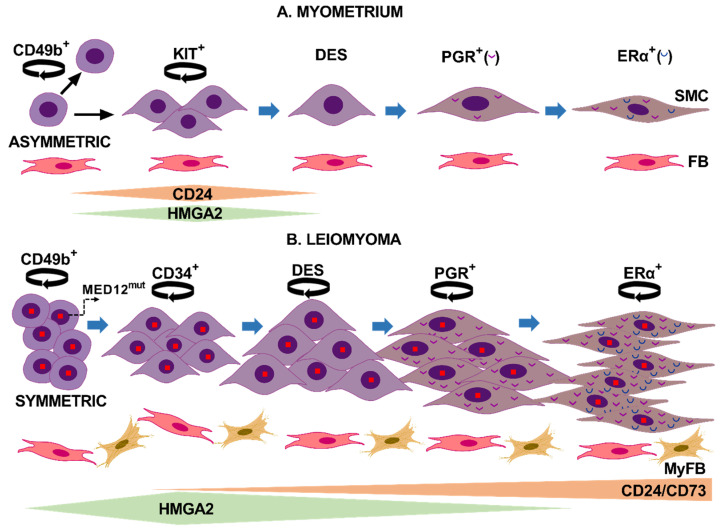
Proposed models illustrate cell proliferation and differentiation mechanisms in normal myometrium and uterine leiomyoma. (**A**) In normal myometrium, a CD49b stem cell asymmetrically divides (circle arrow) in two daughter cells. One cell remains a stem cell with unlimited proliferative ability, whereas the other daughter cell acquires the KIT receptor and commits to becoming a smooth muscle cell (SMC). KIT^+^ cells are a transit-amplifying cell population that expresses high levels of HMGA2 and CD24 during cell proliferation, and their expression levels decrease as cells begin to express desmin. In the next stage, cells acquire ovarian receptors expression, first PGR and then ERα. Communication between SMC and fibroblast (FB) may occur during the proliferation and differentiation process. (**B**) In leiomyomas, hypoxia-induced activation of *MED12* mutated CD49b stem cells leads to symmetrical division and stem cell expansion. These stem cells further differentiate to CD34^+^/CD49b^−^ cells expressing CD24 and probably CD73. These last two surface markers remain upregulated during the entire differentiation process to SMC. CD24 cells express increased levels of HMGA2 that remain elevated until PGR greatly increases expression. Further differentiation steps lead to the expression of desmin, then PGR, and finally ERα. Compared to normal cells, mutant leiomyoma cells express higher levels of ovarian hormone receptors. Cells maintain proliferative capacity during the differentiation process, contributing to tumor growth. Fibroblasts differentiate into myofibroblasts (MyFB), and both cell types may interact with LM cells to regulate tumor growth. This figure was created using Microsoft PowerPoint version 2016.

Based on our results and published studies, we propose two different mechanisms of cell proliferation and differentiation for LM and myometrium (Figure 4). In LM, *MED12* mutant CD49b^+^ stem cells may symmetrically divide, which induces expansion of the stem cell pool resulting in the abundant CD49b^+^ cell clusters observed in tumor slices. Then, mutant SSCs divide to form progenitor cells that proliferate and differentiate, promoting slice repopulation in vitro and tumor mass in vivo [23]. Sustained cell division is evidenced in tumor slices by the wide expression of the Ki67 nuclear marker. This hierarchical mechanism of stem cell proliferation is common during wound healing and regeneration and can be induced in cancer stem cells after an appropriate stimulus [23,24].

In mice myometrium, a classical pulse-chase experiment for in vivo bromodeoxyuridine (BrdU) labeling of cells showed BrdU c-Kit^+^ cells adjacent to SSC. The authors suggested that c-Kit^+^ cells may originate from stem cells after asymmetric division as a transient-amplifying cell population for subsequent terminal differentiation [25]. In agreement with this hypothesis, the detection of CD49b cells dispersedly through all myometrium slices supports a continuous self-renewal of stem cells in normal tissue, where the KIT^+^ daughter progenitor cells are the only ones capable of controlled expanding and then differentiate (Figure 4).

We established the hierarchy of stem cell differentiation, reinforcing and expanding prior knowledge. For instance, ovarian receptors appear late during stem cell differentiation to smooth muscle cells, while desmin arises early during differentiation [15,26,27]. Thus, we observed that in both normal and tumor slices, ovarian receptor expression gradually increased and PGR expression preceded that of ERα. The presence of DES^−^/CD24^+^ cells indicates that the CD24 marker appears before desmin expression. Interestingly we could colocalize CD24 and CD34 markers in a few LM cells, indicating that CD24 expression may arise in the intermediary CD34 cells, supporting CD24 as an early differentiation marker [6]. Taken together, these and previous findings indicate that in LM, CD49b stem cells differentiate to CD24/CD34 intermediary cells that, in a further step, begin to express desmin. Desmin-expressing cells then differentiate to PGR^+^ cells that finally acquire ERα expression (Figure 4B).

CD34 has been identified in myometrial stem/progenitor-like cells [28], but we could not detect the CD34 marker in long-term myometrial slices, possibly for its transient expression. The absence of CD34 marker in normal slices and the inability to colocalize KIT with CD24 prevents knowing the appearance of these markers during myometrial cell differentiation.

During the normal course of skeletal muscle differentiation, *HMGA2* mRNA levels increased after skeletal muscle stem cell activation, remained upregulated during myoblast proliferation, and gradually decreased when myoblasts were induced to differentiate [29]. Similarly, the cell surface glycoprotein CD24 is also expressed in muscle stem/satellite cells, and its expression was associated with muscle fibers regeneration [30,31]. Given the stimulatory role of HMGA2 and CD24 in vitro and in vivo, both markers may play fundamental roles in myometrium and LM growth, even though striking differences were found between the two tissues. Thus, the similar CD24^+^, HMGA2^+^, and KIT^+^ cell numbers in the myometrium slices suggest that both stimulatory molecules are involved in KIT proliferation and differentiation to desmin-expressing cells. Interestingly, we did not find colocalization between CD24 or HMGA2 markers and progesterone receptors in myometrial slices, suggesting that normal cells expressed CD24 and HMGA2 only in the early stages of cell differentiation (Figure 4A). On the contrary, in LM slices, PGR colocalized with both HMGA2 and CD24 (Figure 3B,C). Moreover, the broad expression of CD24 in LM cells at T29 suggests that CD24 expression starts from the intermediary CD34 cell to fully differentiated SMC that express both ovarian receptors (Figure 4B). This result correlates with the abundance of CD24^hi^ cells previously detected in LM [6]. The inability of tumor cells to repress CD24 and the wider expression of HMGA2 during the differentiation process may favor in vivo tumor growth. Supporting this notion, CD24 induced tumor growth, invasion, and metastasis in different cancers [32]. HMGA2 upregulation has been associated with the growth of several mesenchymal tumors, including LM [9,33,34,35,36,37,38,39]. In addition, the 4 tumors analyzed in this study maintained *MED12* mutation while expression of *HMGA2* increased after long-term culture, supporting the coexistence of both alterations in fibroids [10,35]. In this sense, the transcriptional profile of fibroids subtypes demonstrated that the *MED12* and *HMGA2* types shared the most differentially expressed genes and commonly dysregulated pathways [7].

Of note, the CD73 surface marker was detected only in tumor cells and induces tumor growth and metastasis in several solid neoplasms [40]. Other surface markers upregulated in LM include integrin β1 and fibronectin-specific integrin receptor CD51/CD61 [41]. These markers, together with CD24 and CD73, are particularly interesting because they are potential targets for antibody-based therapy, such as injection directly into uterine fibroids of nanoparticles decorated with therapeutic antibodies. Indeed, treatment with CD24 monoclonal antibody reduced tumor burden in mice harboring bladder, pancreatic, lung, ovarian, and colon cancer [32]. Additionally, a β1 integrin antibody decreased leiomyoma cell proliferation in a time- and concentration-dependent manner [41].

The lack of blood vessels to transport O_2_ and the tissue thickness of 500 µm may favor hypoxia in long-term organ culture, a condition widely related to stem cell activation [28,42]. Transplanted leiomyoma and myometrium tissue in nude mice showed reduced size when tissue was inoculated with Matrigel^TM^, which contains multiple angiogenic growth factors and has widely been used to induce angiogenesis in the implanted tissue [43]. On the contrary, the engrafted tissues maintained their original size with reduced vascularity without Matrigel^TM^. Similarly, the increase in vascularized areas within pancreatic cancer xenografts decreased tumor size and significantly reduced the stemness of cancer cells [44]. Therefore, although somatic stem cells rarely divide and maintain a relative quiescence, low oxygen levels in tissue slices after long-term culture may trigger stem cell proliferation and differentiation. Remarkably, these processes occurred without hormonal stimulus, which seems counterintuitive as fibroids are hormone-dependent tumors. However, in a patient-derived xenograft model of LM [45], tumor cells were viable after 10 weeks of xenotransplantation in the absence of E2 and P4. Authors suggested that local factors persist in tissues for an extended period, maintaining cellular pathways active [45]. In this sense, previous studies have demonstrated a signaling cross-talk between IGF-1 and ERα [46]. The binding of IGF1 to IGF1R induces ERα phosphorylation and activation leading to ERα dependent gene transcription in the absence of E2 [46,47]. Target genes include ERα itself and PGR [46,47]. Our results showed increased *IGF1/IGF1R, ESR1* and *PGRA* mRNA levels and a greater number of ERα and PGR cells in long-term LM cultures. IGF1 is a mitogen and a differentiation factor that acts locally via autocrine or paracrine signaling. Given the proven role of IGF/IGF1R in fibroids proliferation [5,45,48,49], our data suggest that IGF1 may activate ERα in a ligand-independent manner, promoting tumor cell proliferation. IGF signaling is regulated by a family of specific IGF-binding proteins (IGFBPs) [50] that we also found differentially regulated in leiomyoma compared to myometrial slices.

## 5. Conclusions

Long-term organ culture maintains the original stem cell niches allowing the analysis of the interactions and signaling processes involved in stem cell proliferation and differentiation. Comparative transcriptome analysis would shed light on the mechanisms involved in normal uterine homeostasis and leiomyoma deregulation, making long-term organ culture a helpful platform for novel therapeutic discovery. Additionally, characterizing the regulation of crucial myometrial genes is essential to understanding normal human birth and obstetric complications, including preterm labor.

## Data Availability

Data is contained within the article and Supplementary Materials.

## Supplementary Materials

The following supporting information can be downloaded at: https://www.mdpi.com/article/10.3390/biomedicines10071542/s1, Figure S1: Cell repopulation of normal and tumor tissue as assessed by hematoxylin and eosin staining; Table S1: Sequence of primer pairs used for qPCR; Figure S2: Proportion of smooth muscle cells and fibroblast after long-term culture and immunostaining of blood vessels; Table S2: List of Antibodies used for immunofluorescence analysis; Figure S3: Driver mutations are maintained in organ cultures at long-term culture; Figure S4: Quantitative PCR data showing differentially expressed genes in leiomyoma and myometrium organ culture.
