# Supplementary material for: Stem Cell Growth and Differentiation in Organ Culture: New Insights for Uterine Fibroid Treatment
Source: Biomedicines. 2022 Jun 29;10(7):1542. doi: 10.3390/biomedicines10071542 (PMC9313456; doi:10.3390/biomedicines10071542)
Supplement: Supplementary file 1 [file biomedicines-10-01542-s001.zip › biomedicines-1772228-supplementary.pdf]

Table S1. Sequence of primer pairs used for qPCR

Gene Name	Forward (5'-3')	Reverse (5'-3')	Size (pb)
**CD24**	CTGCTCCTACCCACGCA	AGAGAGTGAGACCACGAAGAGA	171
**CD34**	GCTTTGCTTGCTGAGTTTGCT	TCGTTTCTGTGATGTTTGTTGTG	212
**CD49 (ITGA2)**	TGCCAGAGGAAGGAAAAGGA	TAACTTTGGACCGCTGGAGA	229
**CD73**	TCCTCTCAATGATGCCGCTT	TTGCGTTGATGAATGGGCGA	161
**ESR1**	GAAAGGTGGGATACGAAAAGACC	GCTGTTCTTCTTAGAGCGTTTGA	163
**HMGA2**	AGAGTCCCTCTAAAGCAGCTCA	CAACTGCTGCTGAGGTAGAAATCG	196
**IGF1**	GCTCTTCAGTTCGTGTGTGGA	GCCTCCTTAGATCACAGCTCC	133
**IGF1R**	CGTGAAGATCCGCCATTCTC	TCACTTCCTCCATGCGGTAA	236
**IGF2**	GGGGCAGTGACAAAAGCAAG	CTCAACCCAAGCAACAGGGA	120
**IGF2R**	ACCAGCAAGGCACAAATCAC	GAGTCATCCACCAAGTAGGCA	250
**IGFBP1**	TGGGACGCCATCAGTACCTA	CTCCTGATGTCTCCTGTGCC	133
**IGFBP2**	GACAAGCATGGCCTGTACAAC	AGAAGAGATGACACTCGGGG	145
**IGFBP3**	CCCTGCCGTAGAGAAATGGA	TTGGTGGTGTAGCCTGGGA	200
**IGFBP4**	CATCATCCCCATCCCCAACTG	GAAGCTTCACCCCCGTCTT	122
**IGFBP5**	CCAATTGTGACCGCAAAGGA	GTCGAAGGTGTGGCACTGAA	152
**IGFBP6**	GGTCTACACCCCTAACTGCG	CTGCGGTTCACATCCTGTGG	189
**KIT**	GACAGGCTCGTGAATGGCAT	GCACATCCACTGGCAGTACA	124
**KLF11**	CCAGGTTGCCGGAAGACCTA	GCGTGACAGCTCATCCGAAC	135
**MED12**	GGCCTCCCGATGTTTACC	GTTGGAACTGATCTTGGCAGG	158
**PGR**	AAGGAGTTGTGTCGAGCTCA	GTTTCACCATCCCTGCCAAT	198
**PGR-B**	TCCCCGAGTTAGGAGACGAGA	CTGTGGCTGTCGTTTGTCCC	90
**PLGA1**	AGTCTGAGGCTTTTTCCATCAGT	GGGTCGTGTGTATGGAGGTG	493
**PUM1**	GACGCTATGGTGGACTACTTCT	TGGAACGCACCTGATGTTCTG	142
**XIAP**	TGGCAGATTATGAAGCACGGAT	AGCCCTCCTCCACAGTGAAAG	136

Table S2. List of Antibodies used for immunofluorescence analysis.

Target	Reference	Dilution used
CD24	Abcam (ab199140)	1:100
CD34	Dako	Ready to use
CD49b	Santa Cruz Biotechnology (sc-74466)	1:50
CD73	Abcam (ab175396)	1:50
Desmin	Dako (IR606-Clone D33)	Ready to use
Erα	Santa Cruz Biotechnology (sc-8002)	1:100
HMGA2	Sigma (SAB2701959)	1:100
Ki67	ABclonal (A2094)	1:100
c-KIT (CD117)	Ventana Medical Systems (790-2951)	Ready to use
PGR	Santa Cruz Biotechnology (sc-166169)	1:100
Vimentin	ThermoFisher (MA514564)	1:1000
Alexa Fluor 488 AffiniPure F(ab')$_2$ Fragment Goat Anti-Rabbit IgG (H+L)	Jackson ImmunoResearch (111-546-003)	1:1000
Alexa Fluor Plus 594. Goat anti-Mouse IgG (H+L) Highly Cross-Adsorbed Secondary Antibody	ThermoFisher (A-32742)	1:1000

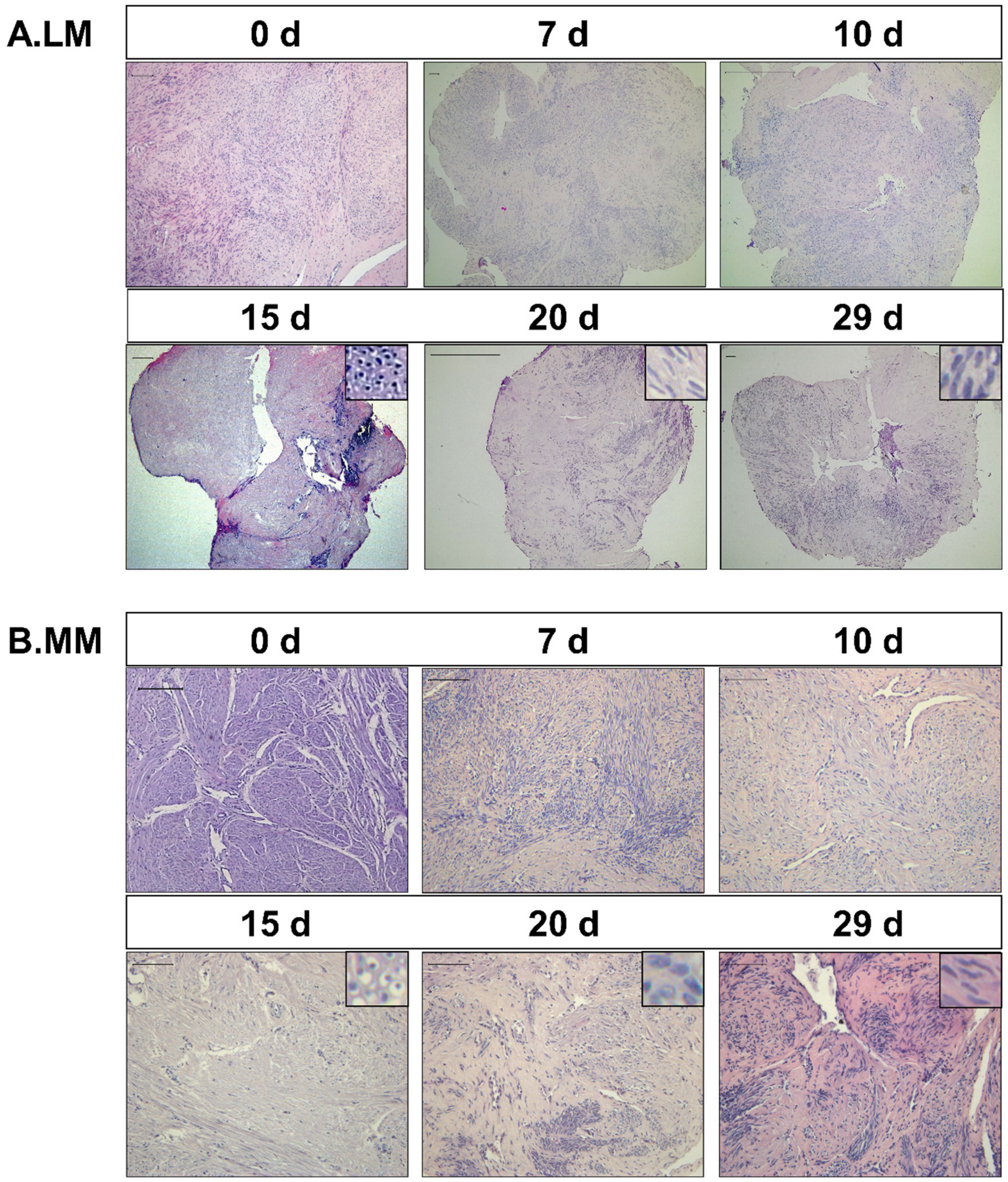

Figure S1. Cell repopulation of normal and tumor tissue as assessed by hematoxylin and eosin staining. Representative images of (A) myometrium (MM) and (B) leiomyoma (LM) organ sections H&E stained at baseline (0 d) and after 7, 10, 15, 20, and 29 days of culture. The inserts (top right) at long incubation times show a higher magnification detail of the cells. Scale bar, 100 µm.

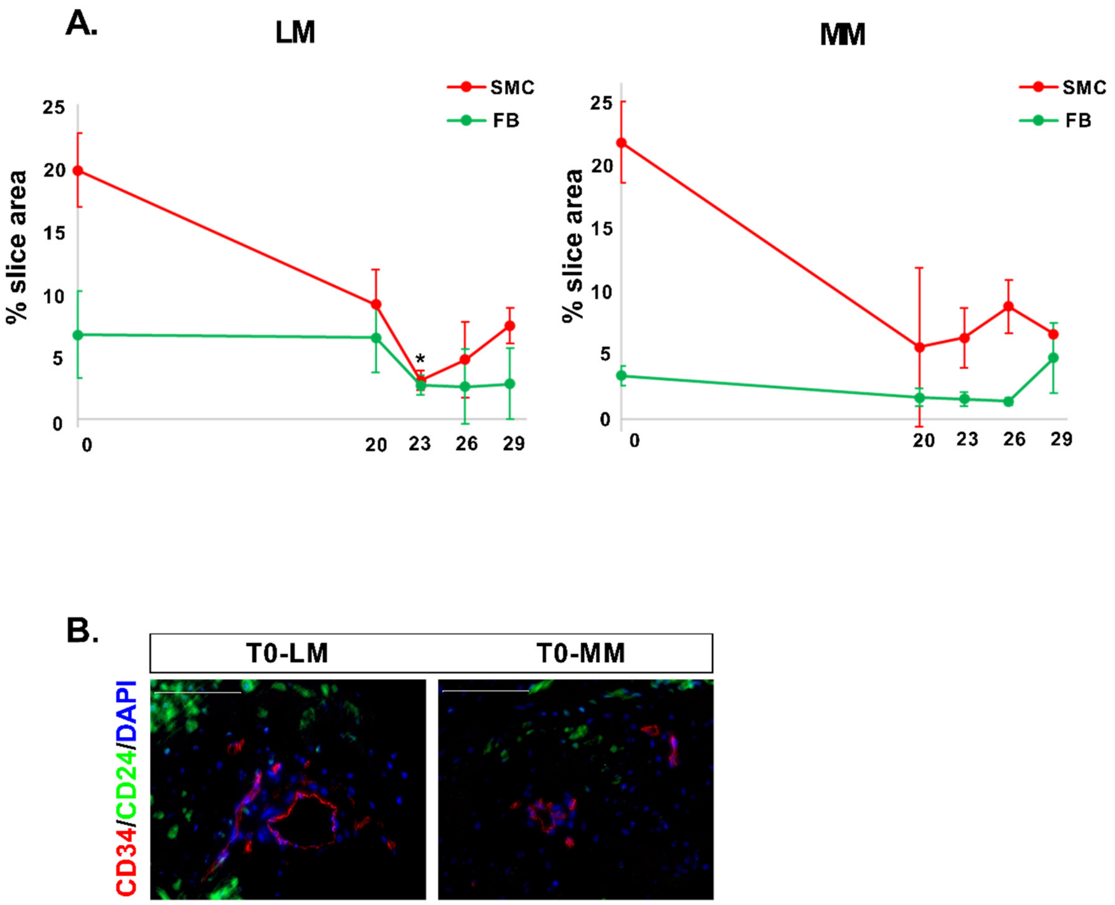

Figure S2. Proportion of smooth muscle cells and fibroblast after long-term culture and immunostaining of blood vessels. (A) Graph represents the percentage of slice area occupied by smooth muscle cells (SMC) and fibroblasts (FB) in leiomyoma (LM) and myometrium (MM) slices at T0 and after 20, 23, 26, and 29 days of culture. For each time point, 3 MM and 3 LM were analyzed. A significant decrease in SMCs was observed in LM after 23 days of culture. Data represent the mean ± S.E.M. *$p<0.05$ compared to control at time zero. (B) IF staining of CD34 (red) and CD24 (green) in leiomyoma (LM) and myometrium (MM) at baseline (T0). Endothelial cells from blood vessels were $CD34^{+}/CD24^{-}$. DAPI stained cell nucleus (blue). Scale bar, 100 µm.

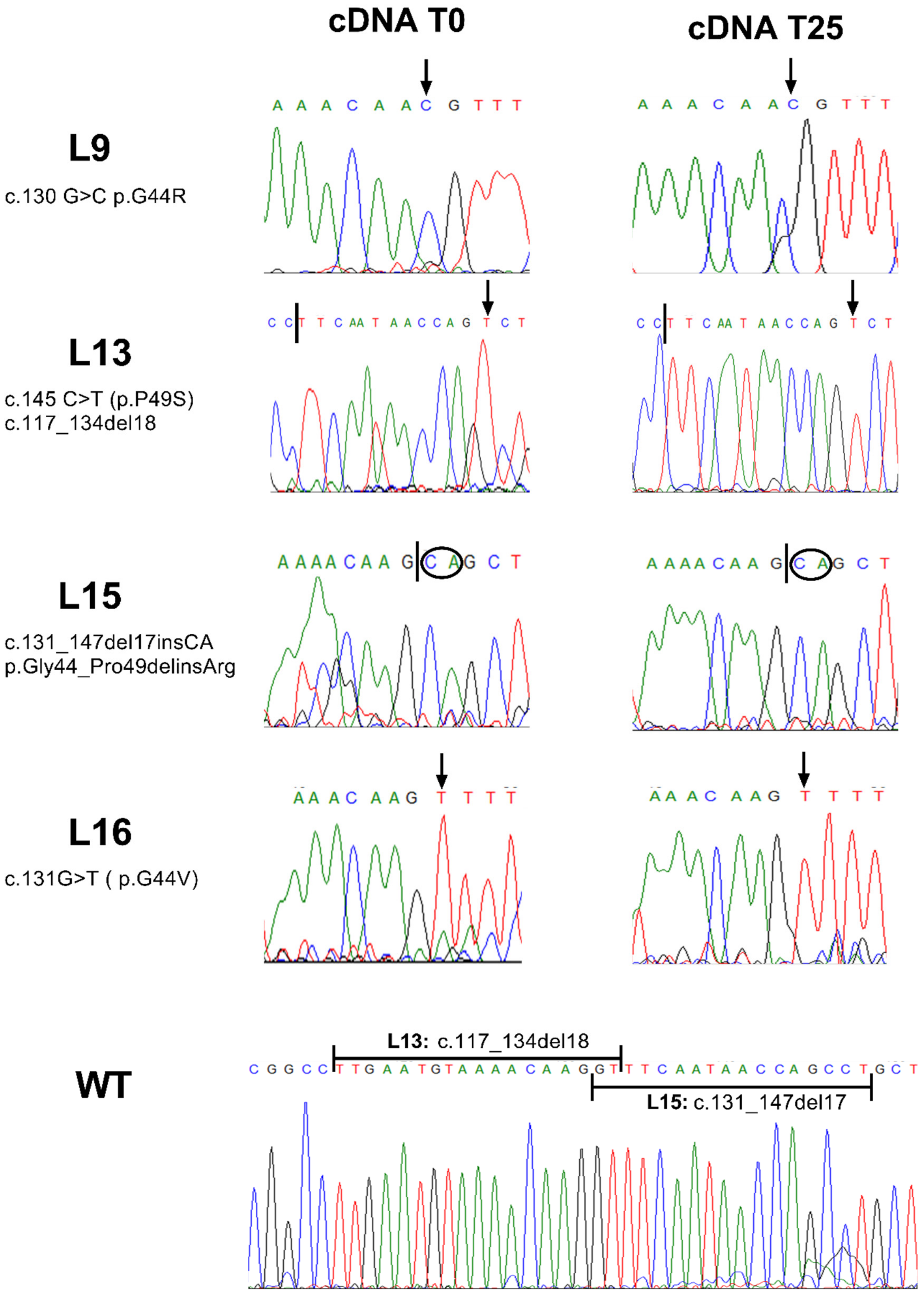
cDNA T0
cDNA T25
L9
c.130 G>C p.G44R
L13
c.145 C>T (p.P49S)
c.117_134del18
L15
c.131_147del17insCA
p.Gly44_Pro49delinsArg
L16
c.131G>T (p.G44V)
WT
L13: c.117_134del18
L15: c.131_147del17

Figure S3. **Driver mutations are maintained in organ cultures at long-term culture**. Sequence electropherograms illustrating different types of somatic mutations in *MED12* in the four leiomyomas (LM). cDNA sequencing demonstrated point mutation in the hot spot codon 44 in two tumors (L9 and L16), one tumor acquired an indel mutation (L15) and the remaining tumor presented a deletion together with a point mutation (L13). Mutant alleles were preferentially expressed at baseline (T0) and after 25 days of culture (T25). The bottom electropherogram represents the wild type sequence (WT) of *MED12* exon 2. Arrows point to single base substitutions. A short vertical black line indicates the position of the nucleotide where deletion starts.

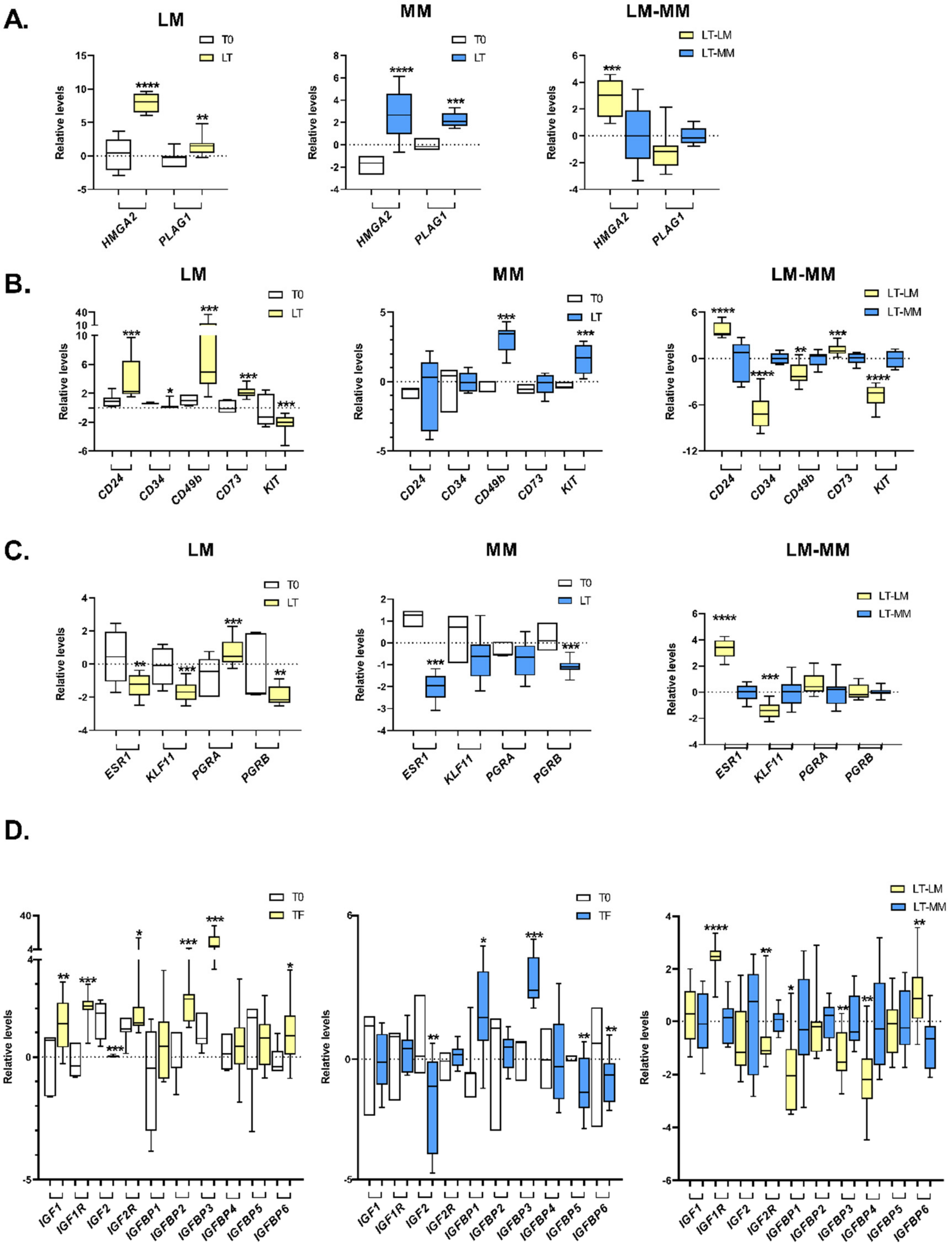
A.
LM
MM
LM-MM
T0
LT
LT-LM
LT-MM
Relative levels
HMGA2
PLAG1
B.
CD24
CD34
CD49b
CD73
KIT
C.
ESR1
KLF11
PGRA
PGRB
D.
TF
IGF1
IGF1R
IGF2
IGF2R
IGFBP1
IGFBP2
IGFBP3
IGFBP4
IGFBP5
IGFBP6

Figure S4. Quantitative PCR data showing differentially expressed genes in leiomyoma and myometrium organ culture.

(A) Box-plot showing relative expression levels of *HMGA2* and *PLAG1* in leiomyoma (LM) and myometrium (MM) organ slices. Long-term culture slices from 4 LMs (16 slices) and 3 MMs (12 slices) displayed significant *HMGA2*, *PLGA1* upregulation compared to their corresponding T0 slices. Increased expression of *HMGA2* was detected in LM compared to MM slices at long-term culture, in agreement with IF data.

(B) Box-plot of relative expression levels of stem cell surface marker (*CD24, CD34, CD73,* and *KIT*) in long-term culture of 16 slices corresponding to 4 leiomyomas (LM) and 12 slices corresponding to 3 myometria (MM) compared to their corresponding T0 (left and middle graphics). Normal and tumor slices were also compared at long-term culture (right graphic).

(C) Box-plot of relative expression levels of *KLF11* transcription factor and progesterone isoforms A (*PGRA*) and B (*PGRB*) and estrogen receptor alpha (*ESR1*) in long-term culture of 4 leiomyomas (LM, 14 slices) and 3 myometria (MM, 12 slices) compared to their corresponding T0 (left and middle graphics). Normal and tumor slices were also compared at long-term culture (right graphic).

(D) Box-plot of relative expression levels of *IGF1, IGF2, IGF1R, IGF2R,* and associated binding proteins *(IGFBP1-IGFBP6)* in long-term culture of 4 leiomyomas (LM, 16 slices) and 3 myometria (MM, 12 slices) compared to its corresponding T0 (left and right graphics). Normal and tumor slices were also compared at long-term culture (bottom graphic). *p<0.1, **p < 0.01, *** p < 0.001, **** p < 0.0001.
